# Depression symptoms and suicide risk of internal medicine residents before and after first year of the COVID-19 pandemic

**DOI:** 10.3389/fpsyg.2023.1074709

**Published:** 2023-08-14

**Authors:** Jose Maria De La Roca-Chiapas, Carlos Francisco Grajeda Gutiérrez, Valeria Judith Íñiguez Venegas, Martha Alicia Hernández González, Verónica Reyes Pérez

**Affiliations:** ^1^Department of Psychology, Division of Health Sciences, University of Guanajuato, Leon, Guanajuato, Mexico; ^2^Mexican Social Security Institute (IMSS), Mexico City, Mexico; ^3^Division of Health Sciences, University of Guanajuato, León, Mexico

**Keywords:** depressive symptoms, cortisol, cholesterol, depression, suicide ideation/risk, internal medical residents

## Abstract

**Introduction:**

Depression is a mental health disorder characterized by the presence of sadness or loss of the ability to feel pleasure, with a high incidence in patients with COVID 19. The investigations have focused on patient care and little on the care of health personnel, these being the ones with the highest mortality rate, so the objective of the study was to investigate the prevalence of depression symptoms and suicide risk and understand the association of depressive disorder and suicide risk with levels of serum cholesterol and low levels of serum cortisol among internal medicine fellows in a specialist medical hospital in Leon, Guanajuato, Mexico, before and after COVID-19.

**Methods:**

In this longitudinal study, internal medicine residents were initially monitored for 2months before starting to care for patients with COVID-19. Participants were asked to fill out depression symptoms and suicide risk surveys. We measured the serum cholesterol and cortisol of each participant, and again after 11months of treating COVID-19 patients.

**Results:**

Depression symptoms and suicide risk were assessed; significant differences were found between the two time periods for depression (*p* < 0.01), and no difference was found for suicide risk (*p* = 0.182). We found a significant correlation between serum cholesterol levels and suicide risk (*r* = 0.366, *p* < 0.01); we also found differences in serum cortisol levels (*p* < 0.01) and cholesterol (*p* < 0.0001) before and after the pandemic.

**Conclusion:**

Caring for patients with COVID-19 in the hospital contributed to an increase in levels of depression symptoms and suicidal ideation, as well as differences in levels of cortisol and cholesterol in resident physicians of internal medicine; among the possible reasons for this change could be the conditions of personal protection while treating patients, the uncertainty in the first months of not knowing how the virus was transmitted and not having or knowing when vaccinations would be available, as well as the lack of a strategy of adequate mental health support from the institutions dedicated to their academic training.

## 1. Introduction

Depression is defined as a common mental health disorder that is characterized by the presence of sadness, loss of interest or pleasure, feelings of guilt, low self-esteem, sleep or appetite disorders, tiredness, and lack of concentration (Saldaña Ibarra and López Ozuna, 2014). The estimated number of people living with depression in the world increased by 18.4% between 2005 and 2015. In Mexico, for the year 2017, it is estimated that depressive disorders were the fourth largest cause of years lived with disability, representing 21.9% of all causes of disability (World Health Organization, 2017).

In a cross-sectional survey (Lai et al., 2020), 1,257 healthcare workers in Wuhan, China, other regions of the province of Hubei, and other provinces with a high incidence of patients with COVID-19, symptoms of depression, anxiety, insomnia, and anxiety were investigated. Of the total, 634 (50.4%) had symptoms of depression, 560 (44.6%) had anxiety, 427 (34%) had insomnia, and 899 (71.5%) had anguish.

Stress levels among medical residents have been found to be higher compared with the general population (Prieto-Miranda et al., 2013). Reports of depression in the High Specialty Medical Unit of the Mexican Institute of Social Security (UMAE-IMSS) range from 25 to 79.6%, with a higher prevalence among second-year residents. It has also been reported that these disorders produce errors in medical prescriptions and the desire to abandon their fellowship; they have also been identified as causes of desertion (Jiménez-López et al., 2015). In a study conducted in 2020 in the UMAE-IMSS No 1 in León, Guanajuato, a greater prevalence of depression was found among second-year students (64.7%), and an association between the diagnosis of depression and suicide risk was identified (*p* < 0.05; González Ramírez et al., 2020).

Many studies have confirmed that biological markers could be related to suicidal tendencies, among which is the level of fats in blood serum, which could play an important role. It is believed that low concentrations of cholesterol in serum also affect the metabolism of serotonin, which leads to depression and deficient control of aggressive impulses, which increases suicide risks. The reduction of total cholesterol in serum is commonly observed among depressed patients that also have depressive symptoms in comparison with healthy controls. In a meta-analysis of 65 clinical trials, with a total of 510,392 participants from 1980 to 2014, it was found that in comparison to non-suicidal patients, suicidal patients had significantly lower total serum cholesterol (Wu et al., 2016).

On the other hand, glucocorticoids influence circadian results during the adaptation of new Zeitgeber signals, including changes in the schedule of light and dark hours and eating times. Therefore, the chronic interruption of the circadian rhythm associated with glucocorticoids may be involved in the physiopathologic adaptation and consequences of the environmental interruption of the circadian synchronization system (Chung and Kyungjin Kim, 2011). It has also been found that cortisol is associated with deficiencies in cognitive control, decision-making, and emotional processing linked with suicidal behavior (Papadopoulou et al., 2017).

In relation to the severe acute respiratory syndrome coronavirus 2 (SARS-CoV-2) pandemic, because of the rapid increase in confirmed cases and deaths, the general population and health professionals experienced psychological problems, such as anxiety, depression symptoms, and stress (González Ramírez et al., 2020). Among the most important repercussions of this pandemic was the impact on the mental health of health workers who treated patients with coronavirus disease 2019 (COVID-19) due to the risk of developing psychological or mental health disorders (Grajeda Gutiérrez, 2022).

Due to these factors and possible relationships, the aim of the study was to investigate the prevalence of depression symptoms and suicide risk and to understand the association of depressive disorder and suicide risk with serum cholesterol levels and low serum cortisol levels among internal medicine residents in a specialized medical hospital in Leon, Guanajuato, Mexico, before and after the first year.

## 2. Materials and methods

We conducted a longitudinal cohort study following the protocol approved with institutional registration number R-2020-1001-018, which measured the depression symptoms, suicide risk, cholesterol, and cortisol of 92 residents of internal medicine at two time points. The first time point was prior to initiating care for COVID-19 patients in the period comprising 3rd to 17th March 2020 (T0). The second time point sought the association of cortisol and cholesterol levels with depressive symptoms and suicide risk among residents of internal medicine from 2nd to 6th March 2021 (T1). We included all internal medicine residents in their second, third, and fourth years (R2, R3, R4) at the UMAE-IMSS. The sample was non-probabilistic for case availability.

### 2.1. Surveys

Beck’s Depression Questionnaire measures the presence and severity of depression symptoms; it is self-administered among adults through 21 responses with a Likert-type scale that ranges from 0 to 3, assessing the intensity of each symptom, and among the Mexican population, it has an Alpha Cronbach of 0.92 (Córdova Osnaya and Rosales Perez, 2011).

Presently, depression and suicide risk can be measured through diagnostic scales that evaluate the presence of these affective disorders through specialized and standardized surveys (Saldaña Ibarra and López Ozuna, 2014). The literature indicates that the suicide rate among doctors is between 28 and 40 per 100,000, and it is higher in comparison with the general population, which is 12.3 per 100,000, indicating that doctors have the highest suicide rate among professionals (Prieto-Miranda et al., 2009).

The Plutchik suicide risk scale was validated in Spain (Santana-Campas and Santoyo Telles, 2018) and is a self-administered questionnaire containing 15 questions with yes/no answers. Each affirmative answer is given 1 point. The total is the sum of the points of all the items. A score equal to or over 6 indicates the presence of suicide risk (Córdova Osnaya and Rosales Perez, 2011; Santana-Campas and Santoyo Telles, 2018).

### 2.2. Blood samples

The blood samples were obtained between 8.30 and 9.30 a.m. after fasting for 8 h and having slept for 6 h. The samples were processed in a clinical analysis laboratory to measure serum cortisol levels by Elecsys Cortisol II electrochemiluminescence immunoassay “ECLIA” using the Cobas 6000 instrument (Roche Diagnostics GmbH, Germany).

### 2.3. Selection criteria

- Internal medicine residents of the UMAE-IMSS who gave informed consent to participate in the study were included.
- Subjects who reported being treated for pathologies that increase cortisol levels (such as Cushing’s syndrome, non-autonomous hypercortisolism, psychiatric disorders, morbid obesity, poorly managed diabetes mellitus, alcoholism) or pathologies in which cortisol is diminished (such as Addison’s, Congenital Adrenal Hyperplasia (CAH) and different disorders related to stress, such as chronic fatigue syndrome, fibromyalgia, and stress disorder), subjects using oral contraceptives, and those with previous psychiatric illnesses were not included.
- Those with incomplete data were eliminated from the study.

### 2.4. Statistical analysis

Previously, we used the Pearson Correlation test to understand the relationship between depression symptoms, suicide risk, and levels of cortisol and cholesterol. To compare depression symptoms, suicide risk, and levels of cortisol and cholesterol before and after 1 year of starting the pandemic, we used the *t*-test of repeated measurements. The data were analyzed with the program SPSS v21; a statistically significant difference was considered to be a *p*-value < 0.05.

## 3. Results

We registered 85 internal medicine residents that were in the second to the fourth year of service and eliminated 32 due to previous psychiatric illness or not completing the questionnaires, leaving a total of 53 subjects for the final analysis.

When comparing depression symptoms using the Beck scale prior to the start of the pandemic with the levels 1 year later, significant differences were found (*t* = 9.618, gl = 52, and *p* < 0.01), with an increase after having treated patients with Covid (see Table 1).

**Table 1:** Psychological tests.

	2020	2021	*p*
Depression symptoms	3.64 ± 3.48	8.83 ± 1.66	0.0001^*^
Suicide risk	3.23 ± 2.99	2.83 ± 2.39	0.182

^*^Statistically significant differences with a value *p* < 0.05.

In the case of suicide risk, when comparing the levels at the beginning of the pandemic with the levels 1 year after the pandemic, significant differences were also found (*t* = 1.35, gl = 52, and *p* = 0.182), with a decrease in the second measurement (see Table 1).

There were 31 men (64.2%) and 22 women (41.5%), of which 34 were in their second year, 16 were in their third year, and 3 were in their fourth year. The values of the laboratory tests are shown in Table 2. Cholesterol, triglyceride, high-density lipoprotein, very-low-density lipoprotein, and cortisol levels were higher one year later, however, low-intensity lipoprotein values were unchanged one year later.

**Table 2:** Laboratory tests.

Laboratory values			
	2020	2021	*p*
Cholesterol	166.5 ± 22.8	178.9 ± 29.9	0.0001^*^
Triglycerides	106.2 ± 46.5	141.6 ± 74.5	0.0001^*^
HDL	47.2 ± 13.2	50.6 ± 13.1	0.015^*^
LDL	98.1 ± 22.8	100.5 ± 35.6	0.489
VLDL	21.2 ± 9.2	27.6 ± 15.4	0.0001^*^
Cortisol	13.0 ± 4.3	11.2 ± 4.1	0.002^*^

^*^Statistically significant differences with a value *p* < 0.05.

We found a general prevalence of depression of 62% (33/53), with the prevalence in women at 72% (16/22) and in men at 54% (17/31). The prevalence of depression by year of residency was 75% for third year residents and 61% for second year residents (see Table 3).

**Table 3:** Prevalence of depression symptoms.

Depressive disorder	
General prevalence	62% (33/53)
Prevalence in women	72% (16/22)
Prevalence in men	54% (17/31)
Prevalence R4	0%
Prevalence R3	75% (12/16)
Prevalence R2	61% (21/34)

For suicide risk, we found a prevalence of 13% (7/53), with the prevalence in women at 13% (3/22), and in men at 12% (4/31) *p* = non significative. By academic year, the prevalence level for suicide was as follows: fourth-year residents, 0% (0/3); third-year residents, 18% (3/16); and second-year residents, 11% (4/34; see Table 4).

**Table 4:** Risk of suicide attempt.

Risk of suicide attempt	
General prevalence	13% (7/53)
Prevalence in women	13% (3/22)
Prevalence in men	12% (4/31)
Prevalence R4	0% (0/3)
Prevalence R3	18% (3/16)
Prevalence R2	11% (4/34)

Additionally, we found a positive correlation between suicide risk and cholesterol levels (*r* = 0.366, *p* < 0.05) but not with serum cortisol levels (*r* = −0.185, *p* = 0.185). We also observed a correlation between cholesterol levels and depression symptoms (*r* = 0.332, *p* < 0.05) but not between cortisol and depression symptoms (*r* = 0.09, *p* = 0.51).

Cortisol serum levels were lower 1 year after the start of the pandemic (*p* < 0.005) and cholesterol levels were greater a year later (*p* < 0.005). We did not find significant differences a year after the pandemic in the results of depression symptoms or suicide risk among the residents.

## 4. Discussion

This study highlights the increase in depression symptoms in the second measurement, the relationship we found between serum cholesterol levels and depression symptoms, and the difference in the risk of suicide compared to pre-pandemic results, levels that are similar to that reported by Wu et al. (2016).

In terms of depression, we estimate that the global prevalence of depression is 5.8% in men and 9.5% in women (Prieto-Miranda et al., 2013). Approximately 60% of these individuals do not receive the necessary help, despite treatment reducing symptoms in more than 50% of cases (Schulz and Arora, 2015). According to estimates, the prevalence of major depressive symptoms varies globally, from 3% in Japan to 16.9% in the United States of America. In other countries, depressive symptoms has a prevalence ranging from 8 to 12% (Kessler et al., 2003). In Mexico, the percentage of women that have symptoms compatible with depression is 5.8%, and for men, it is 2.5% (Saldaña Ibarra and López Ozuna, 2014). During the development of the medical residency, levels of stress have been found to be higher than the general population (Jiménez-López et al., 2015), and by applying diagnostic scales that evaluate the presence of said disorder through specialist questionnaires in residents of the internal medicine service, the prevalence of depression in women was higher, with a global depressive disorder prevalence of 62%. Analysis by gender shows a greater prevalence of cases among women than men, at 72 and 54%, respectively, which coincides with the national statistics that show that more cases of depression tend to be reported among women than among men, contrasted with the 13% obtained in women compared to the 5.8% mentioned previously.

Reports of depression in UMAE-IMSS vary from 26% to 79.6%, with a greater prevalence among second-year residents (Prieto-Miranda et al., 2013). In the present study, we obtained a prevalence of 62%, similar to the prevalence in other studies, with a greater prevalence of depression symptoms in third-year residents, finding 75% of residents followed by second-year residents with 61%. This behavior could be related to factors such as long days in the hospital, work overload, sleep deprivation, complaints from patients and their family members, insufficient medical knowledge, informal learning, under-stimulating academic environments, and high levels of competition (Jiménez-López et al., 2015), as well as the excessive healthcare demand and performing activities inherent of a job in a public health institution. If you add to this the context of an ongoing pandemic, taking into consideration that in this hospital, the third-year residents were exposed to patients infected with SARS-CoV-2 for a longer period, this could explain the increase in and prevalence of depression symptoms with respect to the previous year without exposure to the pandemic. In other words, second-year residents had a greater prevalence of this diagnosis, with a lower prevalence among those in higher academic years.

Similarly, in a recent study on the prevalence of depression symptoms according to academic year in the Guadalajara campus of the Tecnologico de Monterrey, a greater prevalence of depression symptoms was found among fifth-year students, who started their clinical rotations and faced the advent of the SARS-CoV-2 pandemic (Ruvalcaba Pedroza et al., 2021). This result would also explain the increase in the prevalence of depression symptoms with respect to the previous determinations at the start of the pandemic.

In a systematic review, shift work was found to have deleterious cardiovascular effects that could be accentuated by dyslipidemia, which most internal medicine residents have; it is associated with elevated levels of total cholesterol and triglycerides and a reduction in HDL cholesterol (Dutheil et al., 2020). This is consistent with the result that we obtained when comparing levels of serum cholesterol prior to and 1 year after the start of the pandemic.

During stress, in the phase of exhaustion, chronic hormonal changes occur, along with hypercortisolism due to the cortisol secreted being more efficient and starting to accumulate in the circulatory system (Duval et al., 2010). This is why, in situations of chronic stress, without an adequate adaptive response, we would expect that levels of serum cortisol remain above a normal value and remain normal or diminish in the presence of an adaptive response or stressor, which is related to the results obtained when comparing the levels of cortisol prior to the pandemic and 1 year after it started.

A recent study (de la Vega Sánchez et al., 2023), which measured suicidal thoughts in health personnel, found that they increased during the pandemic, which we did not find in our study; however, we did find a high level of risk, and this can be compared with the suicidal ideation found by Liang et al. (2022), which in the third wave, presented a prevalence of 12.6%.

One possible cause of the decrease in cortisol in the second measurement is that it was taken in the last days of residency, when the residents were already finishing their medical specialty cycle.

Given that many studies have confirmed that biological markers (van der Heijden et al., 2008; Salloum, 2017) could be related to suicidal tendencies, we decided to correlate levels of depression symptoms with serum levels of cortisol and apply the Plutchik scale for the diagnostic risk of suicide.

A considerable number of investigations have demonstrated that patients with Major Depressive Disorder often exhibit a decrease in high-density lipoproteins and, curiously, the lack of remission of depression symptoms is also associated with low levels of total serum cholesterol and low-density lipoprotein (LDL) cholesterol; this is consistent with the results of the present study. Further to the observed association between different high-density lipoproteins (HDL) and LDL, the levels of constituent apolipoproteins have been associated with depression (Parekh et al., 2017).

Likewise, the most repeatedly validated biomarkers of depression include hypercortisolemia, hypocholesterolemia, reduced brain-derived neurotrophic factor, and increased interleukin 6 (Caruncho and Rivera-Baltanás, 2010). Our results contradict the results of Caruncho and Rivera-Baltanas and what is reported by Segoviano-Mendoza et al. (2018) since we found an increase in depression symptoms related to a decrease in cortisol levels and an increase in serum cholesterol levels. However, they are similar to the results of other studies, such as those of van Sloten et al. (2023), who found that having better cardiovascular health, as measured by normal cholesterol levels, exercising, and having a normal BMI are favorable factors for not experiencing depression symptoms, and those of the study by Lindqvist et al. (2008) which relates variability in cortisol and symptoms of depression.

One element to draw attention to based on the results is the need for a mental health care program for internal medicine professionals and for any medical residency to be available from the time of admission to the residency and to remain available throughout the entire duration of residency, including psychological and psychiatric care and psychoeducation workshops for the management of emotions, supported by both the hospital and the university that is certifying them, as caring for people with depression symptoms and at risk of suicide would reduce the incidence of suicide and depression in this vulnerable population.

### 4.1. Limitations

This study was conducted in a single tertiary care hospital center, which was converted into a COVID-19 care center. Data on depression symptoms and suicide risk were obtained through questionnaires and not by psychiatric or psychological clinical review.

## 5. Conclusion

Based on our results, we found that there was an increase in depression symptoms and cholesterol and a high prevalence of depression symptoms among internal medicine residents of public hospitals in Mexico, which was higher in women, and that this prevalence is possibly associated with the conditions associated with the care of COVID-19 patients.

There was a reduction in levels of serum cortisol and suicide risk and a significant increase in levels of serum cholesterol and depression symptom values with respect to the results obtained prior to the start of the pandemic.

The decrease in cortisol and suicide risk 1 year following the start of the pandemic may be due to the change in the perception of danger; at the point of the first measurement, there were no vaccines available, the impact of the disease was unknown, there were many deaths, and a lack of knowledge about the management of patients with Covid, while at the point of the second measurement, all the doctors were already vaccinated and knew the treatment and fear of contagion and death had decreased.

We believe that it is necessary to implement medical and psychological control tests for all doctors in health institutions, as well as to evaluate other variables, such as the quality of diet, the adaptive response, the serum levels of the adrenocorticotrophic hormone (ACTH), and lifestyle factors to be able to make an intervention in a timely manner and reduce the risk of suicide and depression symptoms, which have a high cost for individuals and institutions. A mental health care model for health system workers and pre-and post-graduate students is necessary to reduce the incidence of depression and suicide.

## Data availability statement

The raw data supporting the conclusions of this article will be made available by the authors, without undue reservation.

## Ethics statement

We conducted a longitudinal cohort study, following the protocol approved with institutional registration number R-2020-1001-018, with the registration number of the committee of ethics and research 11 CEI 003 2018080, committee of the Unidad Medica de Alta Especialidad, No. 1 del Bajio of the Instituto Mexicano del Seguro Social. The patients/participants provided their written informed consent to participate in this study.

## Author contributions

JR-C participated in the design of the study, review of the results, and preparation of the article. CG and VÍ participated in the evaluation and follow-up of the participants. MH participated in the preparation of the final report and the supervision and follow-up of the participating physicians. VR participated in the statistical analysis and the final report. All authors contributed to the article and approved the submitted version.

## Conflict of interest

The authors declare that the research was conducted in the absence of any commercial or financial relationships that could be construed as a potential conflict of interest.

## Publisher’s note

All claims expressed in this article are solely those of the authors and do not necessarily represent those of their affiliated organizations, or those of the publisher, the editors and the reviewers. Any product that may be evaluated in this article, or claim that may be made by its manufacturer, is not guaranteed or endorsed by the publisher.

## Abbreviations

ACTH: Serum levels of adrenocorticotrophic Hormone
COVID-19: Coronavirus disease 2019
HDL: High-density lipoproteins
HPA: Hypothalamic–Pituitary–Adrenal
LDL: Low-density lipoproteins
SARS-CoV-2: Severe acute respiratory syndrome coronavirus 2
UMAE-IMSS: High Specialty Medical Unit of the Mexican Institute of Social Security
